# Orthogonal design-based investigation of dose, time point, and treatment course on the toxicity-efficacy transition of triptolide in collagen-induced arthritis mice

**DOI:** 10.3389/fphar.2025.1622570

**Published:** 2025-08-12

**Authors:** Shuai Huang, Lu Xu, Yunzhe Wu, Kai Chen, Nan Sun, Zhe Feng

**Affiliations:** The First Clinical Medical College, Nanjing University of Chinese Medicine, Nanjing, Jiangsu, China

**Keywords:** triptolide, collagen-induced arthritis, rheumatoid arthritis, orthogonal design, reproductive toxicity

## Abstract

In recent years, the potential application of *Tripterygium wilfordii* Hook f. (TWHF) in the treatment of rheumatoid arthritis (RA) has garnered increasing attention in both academic research and clinical practice. However, the effective use of *rheumatoid arthritis* is limited in clinical practice by its severe toxic side effects. We conducted a comprehensive analysis of the effects of triptolide dose, treatment course, and time point on the clinical efficacy and safety of treating experimental arthritis. This work employed an orthogonal design grouping and three-factor, three-level dose, treatment course, and time point modeling of collagen-induced arthritis (CIA) in female C57BL/6 mice. Using smears from exfoliated mouse vaginal cells, the estrous cycle was observed. Mice blood was tested by the enzyme linked immunosorbent assay (ELISA) for ovarian hormones such as estradiol (E2) and follicle stimulating hormone (FSH), as well as inflammatory markers such as interleukin-6 (IL-6) and interleukin-17A (IL-17A). In CIA mice, triptolide changed serum E2 and FSH levels, the estrous cycle, arthritis scores, IL-6, and IL-17A levels. The inhibitory effect of triptolide on IL-17A was significantly influenced by the time point of administration. For triptolide therapy of CIA mice, a high benefit-low risk dosage schedule is 150 μg/kg/d −23:00–6 weeks. Therefore, in clinical applications, optimizing the TWHF dosing regimen (including dose, time point, and treatment course) may help to minimize ovarian toxicity while retaining therapeutic efficacy.

## 1 Introduction

*Tripterygium wilfordii* Hook.f. (TWHF) is a traditional Chinese herb that was initially listed in the Compendium of Materia Medica. It can also have immunomodulatory effects that include anti-inflammatory and analgesic, anti-rejection, and immunomodulatory effects (Song et al., 2020). Clinically, it is used extensively to treat a variety of autoimmune-mediated inflammatory conditions, such as rheumatoid arthritis (Lv et al., 2015; Zhang et al., 2023), nephrotic syndrome (Tong et al., 2022), and systemic lupus erythematosus (Chen et al., 2023; Hong et al., 2025). Among these, rheumatoid arthritis (RA) is a chronic connective tissue disease that is one of the most prevalent immune-mediated conditions. Clinically, RA is defined by bone erosion, synovial hyperplasia, and chronic inflammation of the joint tissues. Uncontrolled active RA is characterized by the breakdown of bone and cartilage, progressive disability, and a catastrophic decline in quality of life (Minisola et al., 2021; Adami et al., 2019; Wang S. et al., 2018). TWHF and its several preparation medications (such as Tripterygium polycoride tablet, Tripterygium tablet, and Tripterygium total terpenoids tablet, etc.) are indispensable in the treatment of RA. TWHF extract has been officially recommended in Chinese clinical guidelines for rheumatoid arthritis (RA) since 2004 (Lin et al., 2021), primarily as an adjunct therapy for patients with inadequate response to conventional disease-modifying anti-rheumatic drugs (DMARDs). However, based on clinical observation, the prevalence of RA varies from 0.18% to 1.07% in various populations, with a higher prevalence in females than males (Alamanos and Drosos, 2005), When TWHF was used clinically, the rate of adverse events was 26.7%, with reproductive harm accounting for 11.7% of cases (Zhang et al., 2016). Triptolide was shown to be reproductively harmful to female mice by lowering ovarian and uterine weights, reducing estrogen levels, and lengthening the estrous cycle (Liu et al., 2011; Zhang et al., 2012a; Zhang et al., 2012b). Consequently, research on preventing and managing female reproductive toxicity in RA treatment has become critically important, now representing a key focus in studies on drug toxicity mitigation.

Triptolide is the primary poisonous and physiologically active ingredient in TWHF (Zhou et al., 2012). It has recently been shown in research that triptolide is an anti-RA candidate. According to these investigations, triptolide can lessen the severity of RA and lower serum levels of inflammatory cytokines in rats used as CIA models (Wang S. et al., 2018; Zhang et al., 2024; Li et al., 2020). One of the most popular experimental animal models of RA is the CIA mouse model, which replicates the immune-mediated joint inflammation and bone loss of clinical RA and is used extensively for pathogenesis research, medication screening, and treatment evaluation (Js et al., 2017). In a C57BL/6 mouse model of collagen-induced arthritis (CIA), triptolide has been demonstrated to considerably lower the inflammatory response and improve bone eroding symptoms (Huang et al., 2023). Therefore, the current investigation evaluated the ovarian toxicity and therapeutic efficacy of triptolide in CIA mice using a collagen-induced CIA animal model in female C57BL/6 mice.

Because RA is a chronic autoimmune disease that can last a lifetime, people who have it usually need lifelong or even prolonged therapy. The effectiveness of commonly used anti-rheumatic medications, such as methotrexate, leflunomide, and glucocorticoids, typically takes one to 3 months or longer. However, prolonged use of these medications is frequently associated with the emergence of drug resistance and high-risk adverse effects (Faison et al., 2024).

In general, RA is characterized by fluctuations in disease activity throughout the day. The morning is usually when clinical symptoms of joint pain and stiffness that limit function are most acute, with symptoms waning in the afternoon and evening (Straub and Cutolo, 2007). One of the inflammatory markers (such as IL-6) peaks at night and in the early morning. This pattern of symptoms is controlled by a complex interaction between immune system activation, hormonal changes, and circadian rhythms (Cutolo et al., 2003). The mouse CIA model also exhibits this corresponding circadian fluctuation of cytokines (Li et al., 2004; Ma et al., 2024). Chronotherapeutics is the practice of administering medications in accordance with biological cycles in order to maximize therapeutic results and/or manage side effects. Treatment is given in accordance with circadian cycles, which is a potential therapeutic method. This chronotherapy has demonstrated potential in a number of therapeutic domains, such as the treatment of bronchial asthma, allergic rhinitis, and hypertension (Buttgereit et al., 2013). In the treatment of RA, chronotherapy demonstrates promising therapeutic potential. Studies have shown that the Janus kinase family of protein tyrosine kinases (JAKs) inhibitor Baricitinib, when administered using a chronotherapeutic approach, can specifically modulate the secretion of inflammatory cytokines in CIA mice, thereby significantly enhancing drug efficacy while reducing the required dosage to achieve equivalent therapeutic effects (Yaekura et al., 2020; Takeuchi et al., 2019). Generally speaking, the concurrent therapeutic efficacy is an important concern for others, even though toxicity can be reduced by regulating low dosages of TWHF given and the length of treatment. At the transcriptional-translational level, auto-regulatory feedback loops of internal timing systems are observed in life forms, resulting in distinct gene networks that fluctuate around the clock. We refer to these internal timers as “biological clocks” (Dunlap, 1999). The biological clock gives the host precise timing and strong environmental adaptations by combining internal physiology with external environmental changes. Disrupted or misaligned circadian rhythms can have negative health effects and raise the risk of conditions like cancer, sleep problems, cardiovascular disease, and metabolic disorders (Ruan et al., 2021). In mammals, circadian rhythms are intimately related to sexual behavior, hormone secretion, and reproductive cycles. Both physiology and pathology are impacted by the pervasive regulation of circadian rhythms. Targeting circadian rhythms with temporal treatments offers novel approaches to treating human diseases through biological clock manipulation in the fields of disease intervention and drug discovery. There are currently efforts to use circadian logic in the treatment of RA, methotrexate chronotherapy is used in the treatment of collagen-induced rheumatoid arthritis in rats (Wang X. et al., 2018), a modified-release formulation of prednisone is in clinical use (Buttgereit et al., 2013), and dietary rhythms play a significant role in controlling inflammatory rhythmicity in RA through associated gut microbiota (Ma et al., 2024).

Given these findings, we decided to investigate the toxicity and effectiveness of triptolide across a variety of dosage ranges, administration various points, and treatment courses to systematically elucidate the clinical safety profile and potential therapeutic applications of TWHF.

## 2 Materials and methods

### 2.1 Material

Triptolide (purity >97%) was purchased from Guilin Sanleng Biotechnology Co., Ltd. (Guilin, China). Sodium carboxymethyl cellulose was purchased from Yuanye Biotechnology Co., Ltd (Shanghai, China). Chicken type II collagen (CII) and Freund’s complete adjuvant (CFA) were purchased from Chondrex Company (Washington State, DC, United States). Interleukin-6 (IL-6), interleukin-17A (IL-17A), estradiol (E2), and follicle stimulating hormone (FSH) detection kits were obtained from EnzymeLinked Biotechnology Co., Ltd. (Shanghai, China).

### 2.2 Animals

Female C57BL/6 mice (6–8 weeks) were purchased from Nanjing Qinglongshan Biotechnology Co. The mice were kept in the Nanjing University of Chinese Medicine’s Animal Experiment Center, provided with enough food and water, and had set light and dark times (12/12, lights on from 7:00 (ZT0) to 19:00 (ZT12)). All studies were carried out in accordance with the Institutional Animal Care Committee at the Nanjing University of Chinese Medicine and the China Council on Animal Care at Nanjing University of Chinese Medicine. (Certificate No. 202412A031).

### 2.3 Establishment of the CIA

**FIGURE 1:**
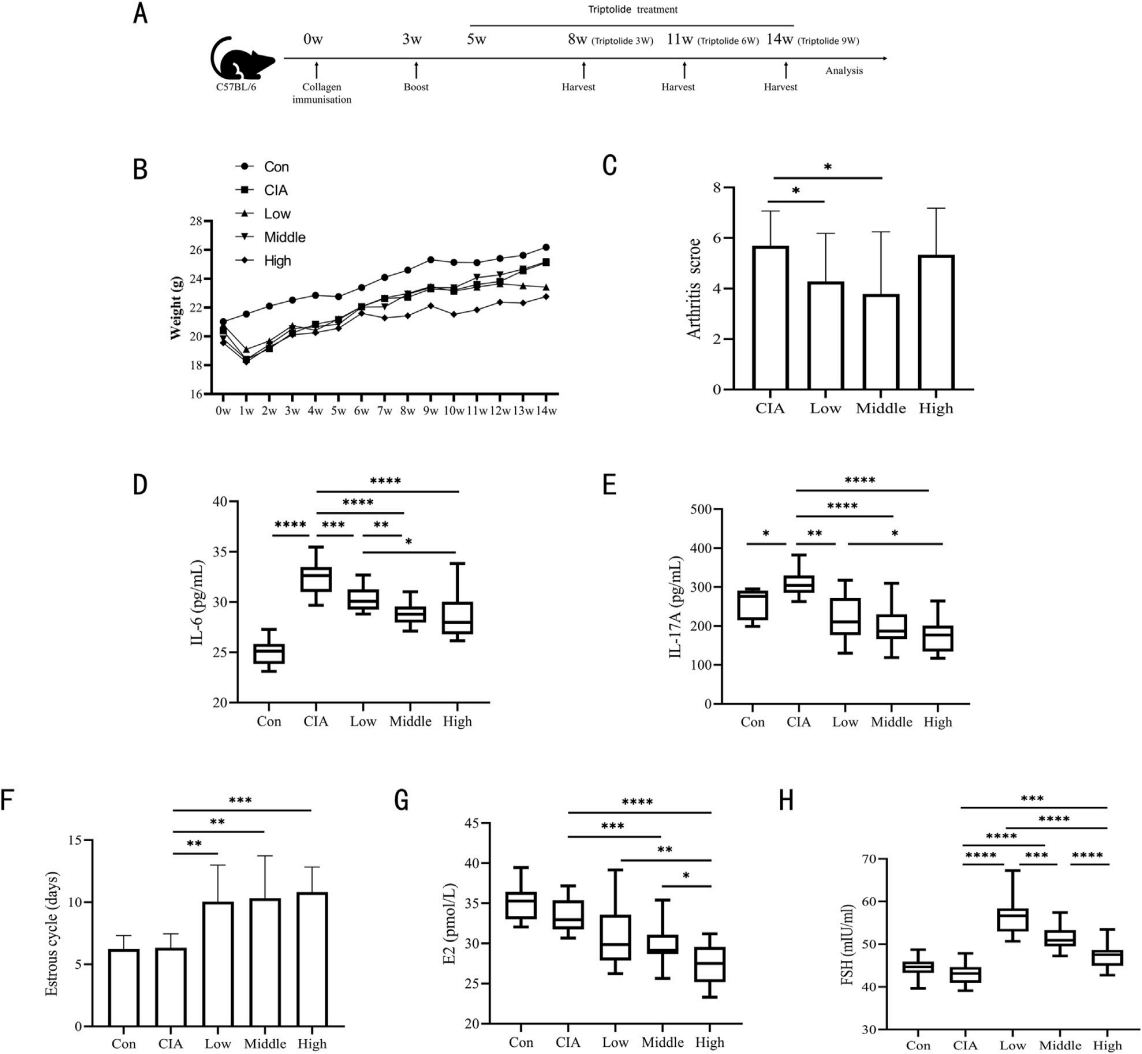
Three triptolide dosages were administered to CIA mice. **(A)** Timeline for CIA models of mice and triptolide therapy; **(B)** Body weights of mice from 0w to 14w (n = 18 mice/group); **(C)** Mice’s arthritis scores for different triptolide dosages (n = 18 mice/group); **(D,E)** Serum cytokine levels of IL-6 **(D)** and IL-17A **(E)** were measured by ELISA (n = 18 mice/group); **(F)** 14 days before the experiment’s conclusion, the length of the estrous cycle was measured in each mouse group receiving varying doses of triptolide (n = 18 mice/group); **(G,H)** Serum cytokine levels of E2 **(G)** and FSH **(H)** were measured by ELISA (n = 18 mice/group); (Low dose: 50 μg/kg/d; middle dose: 150 μg/kg/d; high dose: 450 μg/kg/d. *, p < 0.05; **, p < 0.01; ***, p < 0.001; ****, P < 0.0001).

We established the experimental arthritis mouse model as described previously (Kai et al., 2006) C57BL/6 mice were immunized with complete Freund’s adjuvant (CFA with *M. tuberculosis*, 2 mg/mL) and chicken type II collagen (2 mg/mL) emulsion twice by intradermal injection (0.1 mL per mouse, the final chicken type II collagen content was 1 mg/mL) on day 0 and 21 (Figure 1A).

### 2.4 Clinical assessment of CIA

The model mice were observed beginning at day 28 after the first injection. Two independent observers with no knowledge of the treatment protocol evaluated the severity of CIA. The severity of CIA was assessed as follows (Mukai et al., 2015): 0–4 grade 0 = normal; 1 = mild erythema or swelling of the wrist or ankle or erythema and swelling of 1 digit; 2 = moderate erythema and swelling of the wrist or ankle or more than three inflamed digits; 3 = severe erythema and swelling of the wrist or ankle; 4 = complete erythema and swelling of the wrist and ankle including all digits. Each limb was graded, giving a maximum score of 16. The mice’s arthritis scores were determined by taking the mean of the two observer scores.

### 2.5 Orthogonal experimental design

Mice having arthritis scores of 2 or above were deemed to be CIA modeling results. Following the initial collagen injection, mice with successfully produced CIA models were grouped using the randomized numerical table approach and gavaged beginning on day 35. Using a L9 (3^4^) orthogonal design, the therapeutic efficacy and reproductive toxicity of triptolide in CIA mice were examined. Three influencing factors were divided into three levels (dose: 50 μg/kg/d, 150 μg/kg/d, 450 μg/kg/d; time point of administration: ZT0, ZT8, ZT16; treatment course:3 weeks, 6 weeks, 9 weeks), as shown in Tables 1, 2.

**TABLE 1:** Factors and levels selected.

Factor	A	B	C
Level	Dose (μg/kg/d)	Point	Course (week)
1	50	ZT16	3
2	150	ZT8	6
3	450	ZT0	9

**TABLE 2:** Array of orthogonal compatibility experiments.

Test	Group	A	B	C
1	A1B1C1	50	ZT16	3
2	A1B2C3	50	ZT8	9
3	A1B3C2	50	ZT0	6
4	A2B1C3	150	ZT16	9
5	A2B2C2	150	ZT8	6
6	A2B3C1	150	ZT0	3
7	A3B1C2	450	ZT16	6
8	A3B2C1	450	ZT8	3
9	A3B3C3	450	ZT0	9

A total of six groups, each consisting of six mice, were also created with matching treatment schedules for blank control groups and model groups (3 weeks, 6 weeks, and 9 weeks).In each group, six mice received a daily gavage of 0.5% sodium carboxymethyl cellulose solution at ZT0, ZT8, and ZT16 (two mice were treated at each time point).

In general, The three main groups of the experiment were the blank control group, the CIA model group, and the triptolide administration group. The specific organization of the experiment was as follows:

(1) The blank control group consisted of three subgroups (n = 6 each):
  ① Blank control group - end of 3 weeks by gavage: Mice received a daily gavage of 0.5% sodium carboxymethyl cellulose solution for 3 weeks. During each day, at three time points (ZT0, ZT8, and ZT16), two mice that were fixed in the group were gavaged with a 0.5% sodium carboxymethyl cellulose solution.
  ② Blank control group - end of 6 weeks by gavage: Mice received a daily gavage of 0.5% sodium carboxymethyl cellulose solution for 6 weeks. During each day, at three time points (ZT0, ZT8, and ZT16), two mice that were fixed in the group were gavaged with a 0.5% sodium carboxymethyl cellulose solution.
  ③ Blank control group - end of 9 weeks by gavage: Mice received a daily gavage of 0.5% sodium carboxymethyl cellulose solution for 9 weeks. During each day, at three time points (ZT0, ZT8, and ZT16), two mice that were fixed in the group were gavaged with a 0.5% sodium carboxymethyl cellulose solution.
(2) The CIA model group consisted of three subgroups (n = 6 each):
  ① CIA model group-end of 3 weeks by gavage: Mice with CIA modeling success were chosen. Mice received a daily gavage of 0.5% sodium carboxymethyl cellulose solution for 3 weeks. During each day, at three time points (ZT0, ZT8, and ZT16), two mice that were fixed in the group were gavaged with a 0.5% sodium carboxymethyl cellulose solution.
  ② CIA model group-end of 6 weeks by gavage: Mice with CIA modeling success were chosen. Mice received a daily gavage of 0.5% sodium carboxymethyl cellulose solution for 6 weeks. During each day, at three time points (ZT0, ZT8, and ZT16), two mice that were fixed in the group were gavaged with a 0.5% sodium carboxymethyl cellulose solution.
  ③ CIA model group-end of 9 weeks by gavage: Mice with CIA modeling success were chosen. Mice received a daily gavage of 0.5% sodium carboxymethyl cellulose solution for 9 weeks. During each day, at three time points (ZT0, ZT8, and ZT16), two mice that were fixed in the group were gavaged with a 0.5% sodium carboxymethyl cellulose solution.
(3) The triptolide administration group consisted of nine subgroups (n = 6 each), as shown in Table 2:
  ① A1B1C1: Mice with CIA modeling success were chosen. The mice received a daily gavage of 50 μg/kg/d of triptolide solution at ZT16 for 3 weeks.
  ② A1B2C3: Mice with CIA modeling success were chosen. The mice received a daily gavage of 50 μg/kg/d of triptolide solution at ZT8 for 9 weeks.
  ③ A1B3C2: Mice with CIA modeling success were chosen. The mice received a daily gavage of 50 μg/kg/d of triptolide solution at ZT0 for 6 weeks.
  ④ A2B1C3: Mice with CIA modeling success were chosen. The mice received a daily gavage of 150 μg/kg/d of triptolide solution at ZT16 for 9 weeks.
  ⑤ A2B2C2: Mice with CIA modeling success were chosen. The mice received a daily gavage of 150 μg/kg/d of triptolide solution at ZT8 for 6 weeks.
  ⑥ A2B3C1: Mice with CIA modeling success were chosen. The mice received a daily gavage of 150 μg/kg/d of triptolide solution at ZT0 for 3 weeks.
  ⑦ A3B1C2: Mice with CIA modeling success were chosen. The mice received a daily gavage of 450 μg/kg/d of triptolide solution at ZT16 for 6 weeks.
  ⑧ A3B2C1: Mice with CIA modeling success were chosen. The mice received a daily gavage of 450 μg/kg/d of triptolide solution at ZT8 for 3 weeks.
  ⑨ A3B3C3: Mice with CIA modeling success were chosen. The mice received a daily gavage of 450 μg/kg/d of triptolide solution at ZT0 for 9 weeks.

Triptolide was dissolved in a 0.5% sodium carboxymethyl cellulose solution.

### 2.6 Weight

Once a week at a predetermined time, the mice were weighed using an electronic balance with an accuracy of 0.01 g, beginning on the first day of feeding. Prior to weighing, the mice were allowed to feed themselves naturally, and each mouse’s weight was noted separately.

### 2.7 Vaginal smear and estrous cycle

According to the methodology described by Ajayi and Akhigbe (2020). A cotton swab wet with saline was placed into the mice’s vagina at 10:00 every day, rotated gently, and then taken out. Vaginal fluid was placed on glass slides once day for 14 days. The slides were evaluated under a microscope, and images were captured. The phases of the estrous cycle were identified using vaginal cytology: a predominance of nucleated epithelial cells indicated the proestrus stage, the presence of mostly cornified squamous epithelial cells signified the estrus stage, a mix of cornified squamous epithelial cells and leukocytes characterized the metestrus stage, and a predominance of leukocytes marked the diestrus stage.

### 2.8 Enzyme linked immunosorbent assay (ELISA)

The eyeballs of each mouse were removed after isoflurane anesthesia (Clarysse et al., 2023), at the conclusion of the experiment, yielding approximately 1.5 mL of blood. The blood samples were collected and then left to stand for 2 hours at room temperature. To extract the top layer of serum, the blood samples were centrifuged at 4°C, 1,000 g for 15 min. They were then stored at −80°C until needed. IL-6, IL-17A, E2, and FSH levels in serum were determined by ELISA test kits.

### 2.9 Statistical analysis

All the results are expressed as the means ± SD. The orthogonal experimental data were analyzed using SPSS 18.0 via one-way ANOVA. Pairwise comparisons among the control group, CIA model group, and experimental factor groups were conducted using unpaired Student's t-tests in GraphPad Prism 8.0, with a predetermined significance threshold of P < 0.05.

## 3 Results

### 3.1 Toxicity and therapeutic effect of doses

The procedure and schedule for establishing CIA mice and triptolide treatments are shown in Figure 1A. The triptolide administration experimental group used orthogonal experimental design, which is based on the scientific arrangement of multifactorial tests to provide effective multifactorial analysis with the fewest possible experiments (Wiboonsirikul et al., 2024). In order to identify the main influencing elements and the best parameter combinations, the degree of independent influence of each component on the outcomes can be evaluated by computing the mean difference of various values of each factor (main effect analysis) (Rao et al., 2008). Based on the orthogonal experimental design employed in this study, the administration time points and treatment courses of triptolide in the low-, medium-, and high-dose groups were arranged as follows: the low-dose group (50 μg/kg/d) received A1B1C1 (ZT16 for 3 weeks), A1B2C3 (ZT8 for 9 weeks), and A1B3C2 (ZT0 for 6 weeks); the medium-dose group (150 μg/kg/d) received A2B1C3 (ZT16 for 9 weeks), A2B2C2 (ZT8 for 6 weeks), and A2B3C1 (ZT0 for 3 weeks); and the high-dose group (450 μg/kg/d) received A3B1C2 (ZT16 for 6 weeks), A3B2C1 (ZT8 for 3 weeks), and A3B3C3 (ZT0 for 9 weeks).Within a week following the initial collagen injection, all CIA mice showed characteristic weight loss, and after that, there was a trend toward body weight recovery in all CIA mouse groups. After a week of administration, the mice in the high-dose triptolide group had lower body weights than the mice in the other groups, and this difference remained until the end of the experiment. After 7 weeks of dosing, the mice in the medium-dose triptolide group started to lose body weight (Figure 1B). Triptolide administration at varying doses attenuated arthritis scores, with the observed reductions being statistically significant in both low- and medium-dose groups. (Figure 1C). After receiving varying dosages of triptolide, the CIA mouse model had a high degree of resemblance to RA in terms of important pro-inflammatory cytokines. Specifically, CIA mice had much higher levels of IL-6 and IL-17A, while triptolide gavage resulted in significantly lower levels of IL-6 and IL-17A (Figures 1D,E).

The primary symptoms of TWHF’s harm to the female reproductive system are hypogonadism, premature ovarian failure, and irregular or amenorrheic menstruation. Vaginal cytology was performed daily for 14 days prior to sampling to monitor estrous cycles. All triptolide-treated groups exhibited prolonged estrous cycle duration compared to controls (Figure 1F). When we looked at E2 and FSH in mouse serum, we found that following triptolide therapy, FSH was significantly higher and E2 was much reduced. (Figures 1G,H). Among these, serum E2 displayed a dose-dependent decline in E2 expression; it is noteworthy that low-dose triptolide significantly increased FSH, which then gradually decreased at medium and high doses of triptolide. This phenomenon may be closely associated with the feedback regulation mechanisms of the hypothalamic-pituitary-ovarian (HPO) axis.

In the CIA mouse model, the experimental findings demonstrated a dose-dependent “efficacy-reproductive toxicity” biphasic action of triptolide. E2, the most potent and predominant form of estrogen in females, plays a central role in both physiological and pathological processes in women (Hammes and Levin, 2019). Although high-dose triptolide demonstrates significant anti-inflammatory effects (IL-6, IL-17A), it shows limited efficacy in improving arthritis scores and induces severe ovarian dysfunction (E2, estrous cycle), While low-dose triptolide reduces arthritis scores and lowers IL-6 and IL-17A levels, its significant elevation of FSH and disruption of estrous cyclicity render it non-ideal as a therapeutic dosage. Medium-dose triptolide not only significantly reduced arthritis scores and levels of IL-6 and IL-17A, but also caused substantially less ovarian damage (E2, estrous cycles) compared to the high-dose group. Thus, CIA mice treated with a median dose of 150 μg/kg/d showed a modest toxicity response and a decent therapeutic effect.

### 3.2 Toxicity and therapeutic effect of courses

**FIGURE 2:**
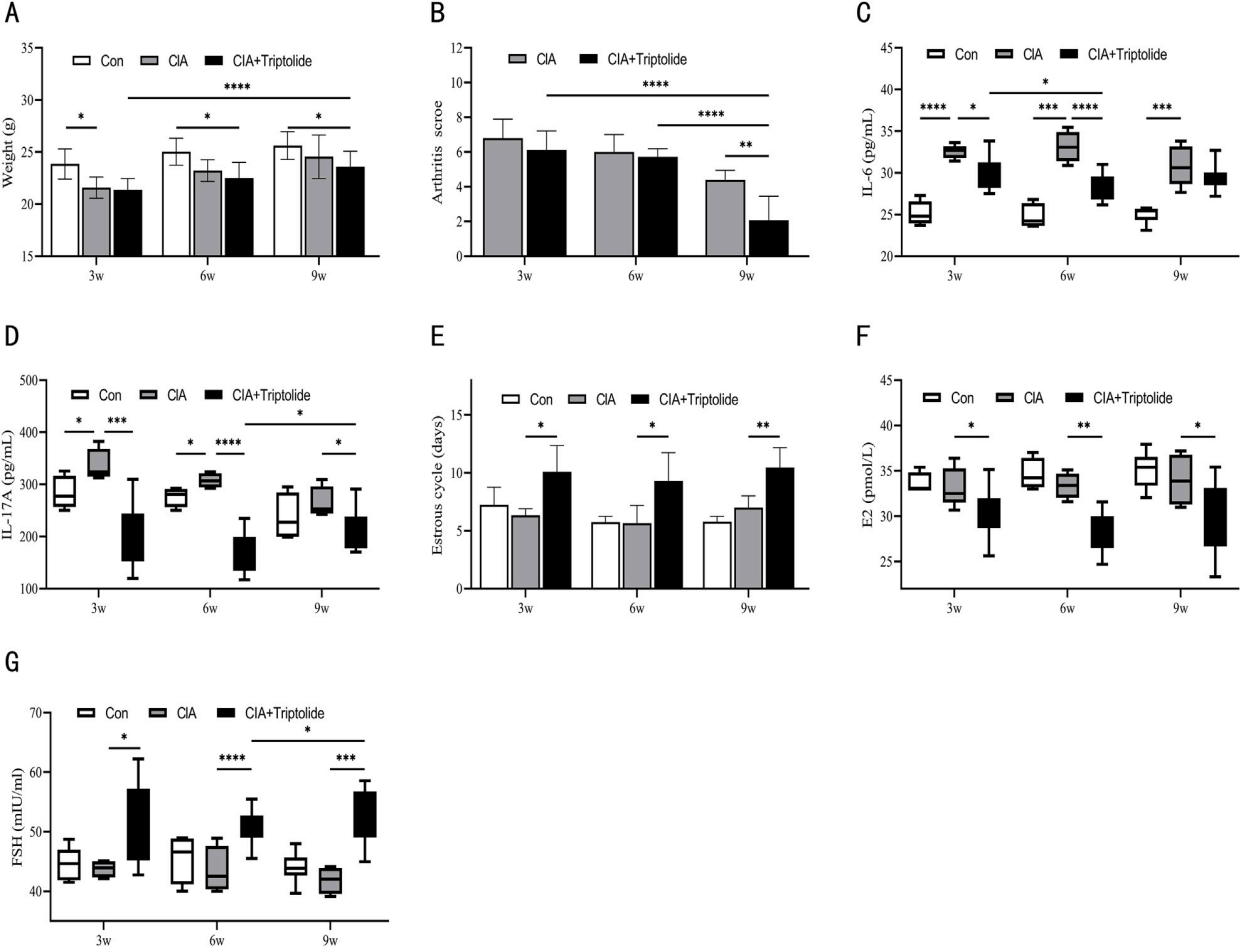
Three triptolide courses were administered to CIA mice. **(A)** Mice’s body weights for different courses (n = 6 in blank control group, n = 6 in CIA model group, n = 18 in CIA + triptolide group); **(B)** Mice’s arthritis scores for different triptolide courses (n = 6 in CIA model group, n = 18 in CIA + triptolide group); **(C,D)** Serum cytokine levels of IL-6 **(C)** and IL-17A **(D)** were measured by ELISA (n = 6 in blank control group, n = 6 in CIA model group, n = 18 in CIA + triptolide group); **(E)** 14 days before the experiment’s conclusion, the length of the estrous cycle was measured in each mouse group receiving varying doses of triptolide (n = 6 in blank control group, n = 6 in CIA model group, n = 18 in CIA + triptolide group); **(F,G)** Serum cytokine levels of E2 **(F)** and FSH **(G)** were measured by ELISA (n = 6 in blank control group, n = 6 in CIA model group, n = 18 in CIA + triptolide group); (triptolide administration was started 5 weeks after mice were modeled, 3w: triptolide administration course was 3 weeks; 6w: triptolide administration course was 6 weeks; 9w: triptolide administration course was 9 weeks; *, p < 0.05; **, p < 0.01; ***, p < 0.001; ****, P < 0.0001).

We next looked into how varied dosing courses (3 weeks, 6 weeks, and 9 weeks) affect the toxicity and efficacy of CIA mice. The dosage and administration time point of triptolide in the 3-, 6-, and 9-week experimental groups were as follows: the 3-week group received A1B1C1 (50 μg/kg/d at ZT16), A2B3C1 (150 μg/kg/d at ZT0), and A3B2C1 (450 μg/kg/d at ZT8); the 6-week group received A1B3C2 (50 μg/kg/d at ZT0), A2B2C2 (150 μg/kg/d at ZT8), and A3B1C2 (450 μg/kg/d at ZT16); and the 9-week group received A1B2C3 (50 μg/kg/d at ZT8), A2B1C3 (150 μg/kg/d at ZT16), and A3B3C3 (450 μg/kg/d at ZT0).The changes in body weight of mice in each group at 3, 6, and 9 weeks of administration are shown in Figure 2A. Compared to other treatment courses and the model control group, a 9-week triptolide regimen demonstrated significantly superior efficacy in reducing arthritis scores in CIA mice (Figure 2B). IL-6 levels started to drop after 3 weeks of triptolide treatment and continued to drop until 6 weeks (Figure 2C). Triptolide administration caused a significant drop in IL-17A after 3 weeks. Interestingly, the inhibitory effect of triptolide was reversed at 9 weeks, when IL-17A increased (compared to 6 weeks, p < 0.05 (Figure 2D). The mice began to exhibit longer estrous cycles, lower E2 levels, and higher FSH levels after 3 weeks of triptolide treatment (Figures 2E–G). As treatment was prolonged to 9 weeks, FSH levels among them were higher than in the 6-week group (Figure 2G).

In terms of dosage courses, a 9-week triptolide course was the most effective in suppressing arthritis scores; however, lengthier courses showed a tendency for IL-17A to rebound. It is noteworthy that the risk of ovarian toxicity has increased dramatically with the 9-week regimen (significantly higher FSH levels compared to the 6-week regimen). Furthermore, we considered that a 6-week treatment reduces ovarian toxicity and inflammatory variables. Consequently, the 6-week dosing regimen maintains therapeutic efficacy and lowers ovarian toxicity in CIA mice.

### 3.3 Toxicity and therapeutic effect of time points

**FIGURE 3:**
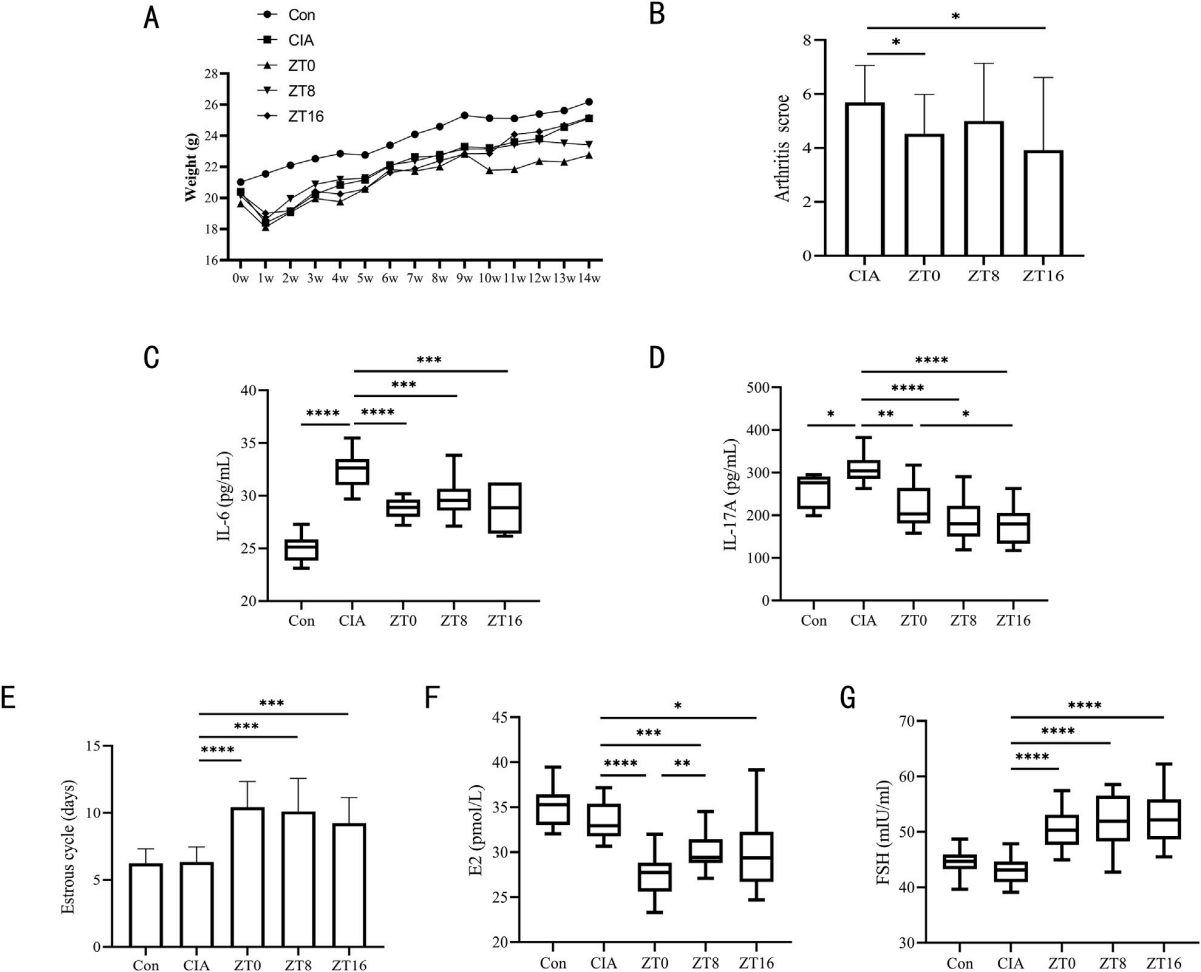
Three triptolide time points were administered to CIA mice. **(A)** Body weights of mice from 0w to 14w (n = 18 mice/group); **(B)** Mice’s arthritis scores for different triptolide time points (n = 18 mice/group); **(C,D)** Serum cytokine levels of IL-6 **(C)** and IL-17A **(D)** were measured by ELISA (n = 18 mice/group); **(E)** 14 days before the experiment’s conclusion, the length of the estrous cycle was measured in each mouse group receiving varying doses of triptolide (n = 18 mice/group); **(F,G)** Serum cytokine levels of E2 **(F)** and FSH **(G)** were measured by ELISA (n = 18 mice/group); (ZT, time after light onset, ZT0: 7:00; ZT8: 15:00; ZT16: 23:00; *, p < 0.05; **, p < 0.01; ***, p < 0.001; ****, P < 0.0001).

We chose three administration time points (ZT, time after light onset) (ZT0, ZT8, ZT16) to assess the effects of these time points on efficacy and toxicity in CIA mice. These time points correspond to morning, midday, and evening, respectively. The administration protocols for triptolide across ZT0, ZT8, and ZT16 experimental groups were designed as follows: the ZT0 group received A1B3C2 (50 μg/kg/d for 6 weeks), A2B3C1 (150 μg/kg/d for 3 weeks), and A3B3C3 (450 μg/kg/d for 9 weeks); the ZT8 group received A1B2C3 (50 μg/kg/d for 9 weeks), A2B2C2 (150 μg/kg/d for 6 weeks), and A3B2C1 (450 μg/kg/d for 3 weeks); while the ZT16 group received A1B1C1 (50 μg/kg/d for 3 weeks), A2B1C3 (150 μg/kg/d for 9 weeks), and A3B1C2 (450 μg/kg/d for 6 weeks).ZT0 mice had lower body weights than the other groups after 4 weeks of triptolide therapy, but ZT8 mice began to lose weight after 7 weeks (Figure 3A). ZT0 and ZT16 mice demonstrated significantly lower arthritis scores (Figure 3B). Following treatment, serum levels of IL-6 and IL-17A were considerably lower at all triptolide time points (Figures 3C,D), and intergroup comparisons revealed that the time of administration did not affect triptolide’s effect on arthritis score and IL-6 reduction in CIA mice (Figures 3B,C). However, IL-17A levels varied according to the time of administration, and triptolide ZT16 treatment had a significantly higher inhibitory effect on IL-17A than ZT0 administration (Figure 3D). Estrous cycle detection in mice revealed that triptolide treatment at various periods delayed the estrous cycle, with no discernible difference between the time points (Figure 3E). Following triptolide therapy, we examined the mice’s serum levels of ovarian hormones and found that FSH expression was much higher and E2 levels were significantly lower (Figures 3F,G). According to the data, triptolide treatment at different time points resulted in varying degrees of suppression of E2 levels in serum; the inhibitory impact of triptolide ZT0 on E2 was more noticeable (Figure 3F). At all time points, however, there was no discernible variation in FSH (Figure 3G).

Compared to the CIA model control group, triptolide administration significantly reduced arthritis scores regardless of morning or evening dosing. However, time-point comparisons revealed that evening dosing specifically led to a more pronounced reduction in IL-17A levels—a key pro-inflammatory cytokine in RA. There was no statistically significant change in FSH levels or estrous cycle at other dosage time points, although morning injection demonstrated the most reproductive damage (significantly suppressed E2 levels). Based on the observed reductions in IL-17A and E2 levels, nighttime administration appears to be the optimal time point.

### 3.4 Interactions between dose, course, and time point on the toxicity and therapeutic effect

In CIA mice, the three variables of triptolide dose, time point of administration, and dosing course exhibited intricate relationships that collectively controlled both the therapeutic benefit of triptolide and the process of reproductive toxicity transformation. Table 3 displays the experimental groups’ weight, arthritis score, and estrous cycle. According to Table 4 findings, the A2B1C3 combination treatment regimen (150 μg/kg/d, ZT16, 9 weeks) most significantly decreased arthritis scores. Regarding the estrous cycle, the effects of triptolide on the mice’s estrous cycle differed depending on the dosing regimens. The combination regimen of A3B3C3 (450 μg/kg/d, ZT0, 9 weeks) significantly prolonged the estrous cycle, whereas the combination regimen of A1B1C2 (50 μg/kg/d, ZT16, 6 weeks) had the least impact. The degree of influence of each element was in the following order for CIA mice’s body weight changes and arthritis scores by triptolide: course > dose > time point; however, for the estrous cycle, the order was dose > time point > course (Table 4). Significant variation in arthritis scores according to the course factor (Table 5). Table 6 displays the levels of IL-6, IL-17A, E2, and FSH in the experimental groups. In terms of inflammatory factor secretion, a high dose (450 μg/kg/d) administered continuously for 6 weeks had the highest inhibitory effect on IL-6 and IL-17A. In the meantime, the triptolide administration time point was highly selective for cytokine regulation: IL-6 was more strongly inhibited by the morning administration (ZT0), whereas IL-17A was more strongly regulated by the nighttime administration (ZT16). Each factor’s degree of influence on IL-6 was as follows: dose > course > time point. Each factor had a varying degree of influence on IL-17A: time point > dose > course (Table 7). Change of the time point factor had a statistically significant impact on the amount of IL-17A. (Table 8).

**TABLE 3:** Experimental results of weight, arthritis score, and estrous cycle based on L9 (3^4^) orthogonal array.

Group	Weight(g)	Arthritis score	Estrous cycle (days)
A1B1C1	21.76 ± 1.16	6 ± 0.71	8.67 ± 1.89
A1B2C3	23.43 ± 0.97	2.71 ± 1.16	10.7 ± 0.98
A1B3C2	23.11 ± 0.99	5.67 ± 0.47	8.25 ± 1.09
A2B1C3	25.18 ± 0.71	0.8 ± 0.98	8.63 ± 1.29
A2B2C2	22.67 ± 1.1	5.75 ± 0.43	9.4 ± 3.01
A2B3C1	21.18 ± 0.76	5.2 ± 0.75	11 ± 1.22
A3B1C2	21.67 ± 1.48	5.75 ± 0.43	10.25 ± 1.79
A3B2C1	21.72 ± 0.86	6.86 ± 0.83	10.25 ± 2.49
A3B3C3	21.6 ± 1.04	3.57 ± 1.4	11.7 ± 1.03

**TABLE 4:** Range analysis of factors of weight, arthritis score, and estrous cycle in L9 (3^4^) orthogonal array.

Source	Weight			Arthritis score			Estrous cycle		
	A	B	C	A	B	C	A	B	C
K1	68.29	68.60	64.66	14.38	12.55	18.06	27.62	27.54	29.92
K2	69.02	67.82	67.45	11.75	15.32	17.17	29.03	30.35	27.90
K3	64.99	65.88	70.20	16.18	14.44	7.09	32.20	30.95	31.03
k1	22.76	22.87	21.55	4.79	4.18	6.02	9.21	9.18	9.97
k2	23.01	22.61	22.48	3.92	5.11	5.72	9.68	10.12	9.30
k3	21.66	21.96	23.40	5.39	4.81	2.36	10.73	10.32	10.34
R	1.34	0.91	1.85	1.48	0.92	3.66	1.53	1.14	1.04

^a^ K1, K2, and K3 are the sum of the first, second, and third levels of this factor, respectively, whereas k1, k2, and k3 are the means of the first, second, and third levels of this factor, respectively. The R is extremum.

**TABLE 5:** Variance analysis of weight, arthritis score, and estrous cycle as indexes.

Source	Weight			Arthritis score			Estrous cycle		
	A	B	C	A	B	C	A	B	C
SS	3.07	1.31	5.12	3.31	1.34	24.75	3.67	2.21	1.67
df	2	2	2	2	2	2	2	2	2
MS	1.53	0.66	2.56	1.65	0.67	12.38	1.84	1.10	0.84
Fvalue value	0.92	0.40	1.54	4.34	1.75	32.51	0.94	0.57	0.43
Pvalue	0.520	0.717	0.393	0.187	0.363	0.030*	0.514	0.638	0.699

^a^ SS: sum of squares; df: Degree of Freedom; MS: mean square; *, p < 0.05.

**TABLE 6:** Experimental results of IL-6, IL-17A, E2, and FSH based on L9 (3^4^) orthogonal array.

Group	IL-6	IL-17A	E2	FSH
	(Ug/mL)	(Ug/mL)	(pmol/L)	(mIU/mL)
A1B1C1	31.13 ± 0.22	194.79 ± 54.18	34.21 ± 3.34	60.4 ± 2.35
A1B2C3	30.19 ± 1.21	228.09 ± 45.49	31.05 ± 2.7	56.35 ± 1.75
A1B3C2	29.46 ± 0.39	233.46 ± 60.34	27.84 ± 1.14	52.26 ± 1.81
A2B1C3	29.06 ± 0.34	198.63 ± 22.49	29.87 ± 2.92	53.24 ± 1.98
A2B2C2	28.7 ± 1.31	175.48 ± 37.54	29.67 ± 1.45	50.71 ± 1.51
A2B3C1	28.53 ± 0.75	231.78 ± 52.15	28.78 ± 2.25	51.37 ± 3.42
A3B1C2	26.39 ± 0.25	144.9 ± 28.43	27.66 ± 2.52	49.12 ± 2.35
A3B2C1	30.35 ± 2.17	172.29 ± 35.54	29.01 ± 0.92	43.99 ± 1.36
A3B3C3	28.22 ± 1.06	207.44 ± 34.35	25.11 ± 1.36	46.68 ± 1.44

**TABLE 7:** Range analysis of IL-6 and IL-17A factors in L9 (3^4^) orthogonal array.

Source	IL-6			IL-17A		
	A	B	C	A	B	C
K1	90.78	86.58	90.02	656.34	538.32	598.86
K2	86.29	89.24	84.54	605.89	575.85	553.84
K3	84.96	86.21	87.47	524.63	672.69	634.16
k1	30.26	28.86	30.01	218.78	179.44	199.62
k2	28.76	29.75	28.18	201.96	191.95	184.61
k3	28.32	28.74	29.16	174.88	224.23	211.39
R	1.94	1.01	1.82	43.90	44.79	26.77

^a^ K1, K2, and K3 are the sum of the first, second, and third levels of this factor, respectively, whereas k1, k2, and k3 are the means of the first, second, and third levels of this factor, respectively. The R is extremum.

**TABLE 8:** Variance analysis of IL-6, IL-17A as indexes.

Source	IL-6			IL-17A		
	A	B	C	A	B	C
SS	6.20	1.82	5.00	2944.19	3204.19	1080.38
df	2	2	2	2	2	2
MS	3.10	0.91	2.50	1472.09	1602.09	540.19
Fvalue	2.37	0.70	1.91	18.36	19.98	6.74
Pvalue	0.297	0.590	0.343	0.052	0.048*	0.129

^a^ SS: sum of squares; df: Degree of Freedom; MS: mean square; *, p < 0.05.

E2 secretion was significantly suppressed by the high dose, morning administration (450 μg/kg/d, ZT0) in terms of triptolide ovarian estrogen levels; however, E2 was less influenced by the low dose, evening administration (50 μg/kg/d, ZT16). Each factor’s impact on E2 levels is as follows: dose > time point > course, with triptolide dose and time point having a statistically significant difference in serum E2 secretion levels. Regarding FSH, there was low toxicity in the A3B3C2 combination regimen (450 μg/kg/d, ZT0, for 6 weeks), while FSH increased significantly in the A1B1C3 combination regimen (50 μg/kg/d, ZT16, 9 weeks). The order of each factor’s degree of influence on FSH was dose > time point > course (Tables 9, 10).

**TABLE 9:** Range analysis of factors of E2, FSH in L9 (3^4^) orthogonal array.

Source	E2			FSH		
	A	B	C	A	B	C
K1	93.09	91.74	92.00	169.02	162.76	155.76
K2	88.33	89.73	85.16	155.31	151.06	152.09
K3	81.78	81.73	86.03	139.79	150.31	156.27
k1	31.03	30.58	30.67	56.34	54.25	51.92
k2	29.44	29.91	28.39	51.77	50.35	50.70
k3	27.26	27.24	28.68	46.60	50.10	52.09
R	3.77	3.33	2.28	9.74	4.15	1.39

^a^ K1, K2, and K3 are the sum of the first, second, and third levels of this factor, respectively, whereas k1, k2, and k3 are the means of the first, second, and third levels of this factor, respectively. The R is extremum.

**TABLE 10:** Variance analysis of E2, FSH as indexes.

Source	E2			FSH		
	A	B	C	A	B	C
SS	21.51	18.67	9.23	142.56	32.51	3.47
df	2	2	2	2	2	2
MS	10.76	9.33	4.62	71.28	16.26	1.73
Fvalue	24.28	21.07	10.42	10.35	2.36	0.25
Pvalue	0.040*	0.045*	0.088	0.088	0.298	0.799

^a^ SS: sum of squares; df: Degree of Freedom; MS: mean square; *, p < 0.05.

## 4 Discussion

Although contemporary medical research has largely confirmed TWHF as a traditional Chinese medicine for the treatment of RA (Zhang et al., 2021; Feng et al., 2022), its clinical application is constantly surrounded by controversy regarding the “risk-benefit” trade-off because of its severe toxic side effects (Li et al., 2014). The TWHF is presently being studied for “co-administration, regimen limitation, alternative regimens, structural modification, and targeted delivery” in order to reduce risk and increase benefits (Ren et al., 2021; Shan et al., 2023; Wang J. et al., 2018). In order to maximize effectiveness and minimize side effects, chronotherapeutics optimizes the timing, dose, and therapeutic approach of drug delivery by biological rhythms (such as seasonal and circadian rhythms). There is a lot of potential to use temporal aspects in disease intervention and drug development for the treatment of human diseases, and temporal therapeutic techniques that target circadian rhythms as a therapeutic strategy show considerable promise for clinical care.

This study adopted a three-factor, three-level orthogonal experimental design to thoroughly examine the reproductive toxicity and therapeutic effects of various triptolide delivery regimens on female CIA mice. In female CIA mice, we explored the concurrent reproductive damage in mice as well as the therapeutic effects of three triptolide administration doses (50 μg/kg/d, 150 μg/kg/d, and 450 μg/kg/d), three triptolide administration time points (ZT0, ZT8, ZT16), and three triptolide administration courses (3 weeks, 6 weeks, and 9 weeks). We discovered that triptolide was successful in lowering IL-6, IL-17A, and arthritis scores. It also caused varying degrees of ovarian damage in mice, as shown by longer estrous cycles, lower E2, and higher FSH. For the treatment of female CIA mice with triptolide, an optimal dosage schedule of 150 μg/kg/d-23:00–6 weeks is advised based on the balance of toxicity and efficacy. This regimen lowers the risk of reproductive toxicity while maintaining therapeutic efficacy comparable to higher doses. The analysis of this study showed that the effects of triptolide dose, course, and time point varied on various observables: the triptolide course had a significant impact on body weight and arthritis scores, and the triptolide dose dominated many indicators (estrous cycle, IL-6, E2, and FSH). However, the time point of administration had a significant effect on IL-17A variation. The primary cytokine released by Th17 cells, which are crucial to the pathophysiology of RA, is IL-17A. In RA, chronic inflammation and joint deterioration are intimately linked to Th17 cell overactivation and IL-17A overproduction (Gaffen, 2009). The time-dependent action of IL-17A implies that the exact application of temporal therapies may be advantageous for TWHF therapy of RA.

In this study, we looked into how female CIA mice responded to various triptolide doses, courses, and time points. In animal mice, the dose, course, and time point of triptolide did have varying effects on the therapeutic and toxicity differences of arthritis. This confirmed the use of “medium-dose-evening administration” to decrease toxicity and boost efficacy, and it provided experimental support for TWHF to develop personalized RA treatment regimens in the clinic.

This study’s limitations include the fact that various triptolide doses, courses, and time points have varying harmful and therapeutic effects on various target organs. This study did not look at other significant target organ toxicities; it solely evaluated ovarian toxicity and efficacy in female CIA mice. Additionally, the toxicity of triptolide varied with sex. This study also lacks parallel control data on efficacy-toxicity in male mice.

## 5 Conclusion

In this study, we used an orthogonal experimental design to evaluate the effects of triptolide dose, course, and time point on the reproductive toxicity and therapeutic effects in mice with collagen-induced arthritis (CIA). According to the study’s findings, triptolide efficiently reduced pro-inflammatory factor levels and considerably reduced arthritis symptoms, but it also interfered with reproductive function. Using experimental research, the ideal triptolide administration schedule was ultimately established to be 150 μg/kg/d at 23:00 for 6 weeks. This regimen achieved the best possible balance between therapeutic benefit and safety, reduced the risk of reproductive toxicity while maintaining efficacy, and offered a crucial experimental foundation for the widespread and secure use of clinical TWHF preparation in the management of rheumatoid arthritis.

## Data Availability

The original contributions presented in the study are included in the article, further inquiries can be directed to the corresponding author.
